# MRI features of spinal Fluorosis: Results of an endemic community screening

**DOI:** 10.12669/pjms.291.3200

**Published:** 2013

**Authors:** Iftekhar Ahmed, Saba Sohail, Munawwar Hussain, Nazeer Khan, Masood Hameed Khan

**Affiliations:** 1 Iftekhar Ahmed, MBBS, MCPS, FCPS, Professor of Medicine, Dow University of Health Sciences, Karachi, Pakistan.; 2 Saba Sohail, MBBS, MCPS, FCPS, Professor Radiology, Dow Medical College, Dow University of Health Sciences, Karachi, Pakistan.; 3 Munawwar Hussain, MBBS, FCPS, Assistant Professor of Radiology,Dow International Medical College, Dow University of Health Sciences, Karachi, Pakistan.; 4 Nazeer Khan, MSC, MS, PhD (Canada), Director of Research & Professor of Biostatistics, Dow University of Health Sciences, Karachi, Pakistan.; 5 Masood Hameed Khan, MCPS, FCPS, FRCP, PhD, Professor of Medicine, Dow University of Health Sciences, Karachi, Pakistan.

**Keywords:** Endemic fluorosis, Thar Desert, MRI, Spine, Screening

## Abstract

***Objectives:*** Fluorosis is endemic in many parts of the world. However community studies on MRI features of fluorosis are lacking. The aim of this study was to determine MRI features of spinal changes in a community with endemic fluorosis in the *Thar* Desert Pakistan.

***Methodology:*** Randomly selected adults from the Village *Samorindh,* district Tharparker, Sindh, Pakistan, with spinal fluorosis diagnosed on plain x-rays and raised serum fluoride levels were studied from June 2008 to January 2009. MRI was carried out on 0.5 T open magnet MRI system. Features of vertebral body, spinal ligaments, intervertebral disc, facet joints, iliac wings and other incidental findings were noted. Sclerosis was defined as low signal intensity on both T1 and T2 weighted images. Results were described as mean and percentage values.

***Results:*** All the studied 27 subjects complained of back ache without neurological signs. The average age was 43.33 ± 10.45 years; 21 being male (77.8%). The most frequent findings included generalized vertebral sclerosis (24, 88.8%), ligamentum flavum hypertrophy (23, 85%), anterior (20, 74%) and lateral (17, 62.9%) disc herniation, thickened longitudinal ligaments, and narrowing of spinal foramina. Hemangioma was seen in 04(14.8%). The most commonly involved level was L1-2, L4-5 and lower dorsal spine.

***Conclusion:*** Vertebral sclerosis, a combination of premature degeneration with anterior disc herniation and an unusually high frequency of vertebral hemangioma formed the spectrum of MRI findings in subjects with spinal fluorosis having back ache but no neurological findings.

## Introduction

 Excessive fluoride exposure affecting the dental and skeletal tissue is termed Flourosis and is reported from many countries of Asia, Europe and Africa,^1^^-^^7^ with only a single report from Pakistan.^8^

 Flourosis results in an osteocondensation by altering bone mass through effects on skeletal mineralization, impaired bone resorption and ion induced decreased bone strength.^8^ These are all contradictory effects leading to a combination of osteosclerosis, stress fractures, ligamentous calcification, ossification and a radiculomyelopathy owing to mechanical compressive effects.^2^^,^^9^^,^^10^

 Although the radiographic (X-ray) findings of Flourosis are well known,^3^^,^^9^^,^^10^^-^^12^ there is limited reporting of MR features of the flourotic spine comprising occasional case reports and two small case series of 5 cases each. MR scan can serve as a tool for early diagnosis and planning proper surgical intervention in the symptomatic patient by visualizing soft tissue and spinal cord changes and associated abnormalities.

 Although, the differential diagnosis of altered marrow signals representing diffuse low signals on MRI are not specific to fluorosis and can be seen in other conditions like myelofibrosis, mastocytosis, lymphoma, osteopetrosis, osteoblastic metastasis, and Paget’s disease. In patients with compressive myelopathy secondary to ossification of PLL and/or LF, fluorosis should be considered as a possible cause, especially in endemic regions. By early detection with non invasive procedure like MRI which show diffuse low signals on all pulse sequences, we can save the patients from drastic effects like fluorotic myelopathy which require surgical decompression to resolve and fractures.

## Methodology

 The Thar Desert in Pakistan is known to be endemic for Flourosis with drinking water levels of fluorine as high as 7-12 mg/L while the acceptable WHO level is 1.5 mg/L.^13^^,^^14^ During a social service campaign, a village named *Samorindh *was identified in District *Tharparker Umerkot*, Sindh, with interesting characteristics. Underground water was the sole source of drinking water with high fluoride levels as described above. The whole population (just under 1000 in number) did not undergo any migration or influx from other community providing a near perfect static model for evaluating the effects of Flourosis.

**Table-I:** A summary of MRI findings in spinal flourosis (N=27).

*Feature*	*Seen in *	*Percentage out of total*
A -Vertebral body Generalized sclerosis End plate sclerosis Reduced vertebral height Anterior osteophytes Block vertebra spondylolisthesis	27 24 03 02 08 02 01	100% 88.8% 11.2% 7.4% 29.6% 7.4% 3.7%
B - Spinal ligaments LF hypertrophy LF ossification Mild thickening of ALL and PLL Moderate thickening of ALL and PLL	23 01 14 08	85.2% 3.7% 51.8% 29.6%
C - Intervertebral disc status Anterior disc herniation Lateral disc herniation Posterior disc herniation	20 17 nil	74% 62.9% 00
D - Neural foramen narrowing	22	81.5%
E - Facet joint sclerosis	5	18.5%
F - Iliac wing sclerosis	19	70.4%
G - Others Hemangioma ( solitary and multiple)*	04	14.8%

Legend: LF= ligamentum flavum, ALL= anterior longitudinal ligament,

PLL= posterior longitudinal ligament

*=incidental finding

 Clinical, radiologic and laboratory screening of this population was carried out in order to identify the morphology of flourosis. Despite the absence of clinical symptoms, the X-rays appearances were consistent with flourotic changes in spine as described in literature. The aim of this particular study was to identify MRI changes in the dorso- lumbar spine of a community afflicted with endemic flourosis.

## Results

 Twenty seven adult subjects were randomly selected out of the total population. Their mean age was 43.33 ± 10.45 years, ranging from 35 to 80 years. Twenty one (77.8%) was males and 6(22.2%) were females with a male to female ratio of 7:2. All the subjects complained of a vague backache but none had any localizing neurological motor or sensory sign. The mean serum fluoride level of the subjects was 0.7846± 0.20 mg/L. Positive MRI findings were seen in all cases in varying combination as described below and summarized in Table-I.

**Fig.1:**
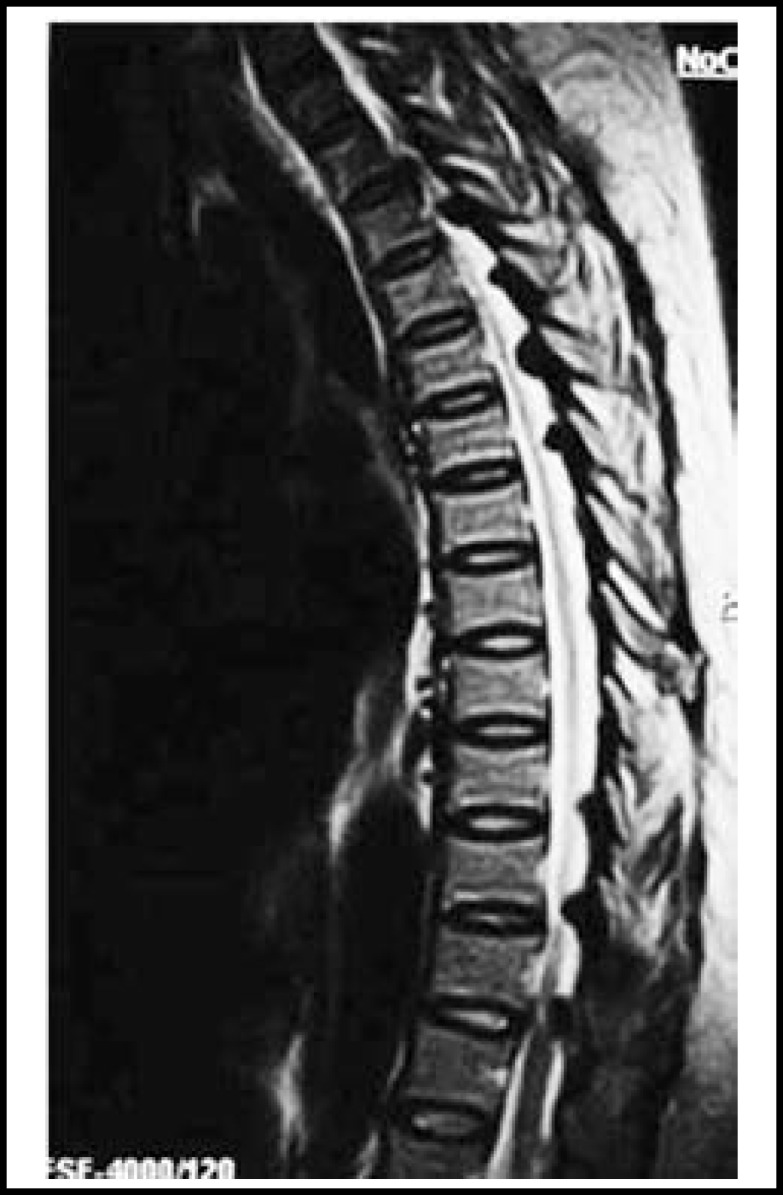
T2 weight MRI of thoracic spine showed thickening of anterior and posterior longitudinal ligaments between T3–T7 and T10–L1 (case 17).

 The vertebrae showed flourotic changes as hypo intense appearance on both sequences (Fig.1). It was diffuse in 24 and restricted to end plate in 03 cases. Twenty five (92.6%) had normal and 2 (7.4%) had reduced vertebral bodies height. Grade I spondylolisthesis was seen in 01 case.

 Anterior osteophytes were seen in 8(29.6%) cases. In 2(25%) cases they were located on two levels and on single level in the rest. The level was L1-L2 in five (62.5%) cases and lower dorsal in 3(37.5%) cases. Single or multiple hemangiomas in the vertebral body was seen in 4(14.8%) cases probably as incidental finding.

**Fig.2:**
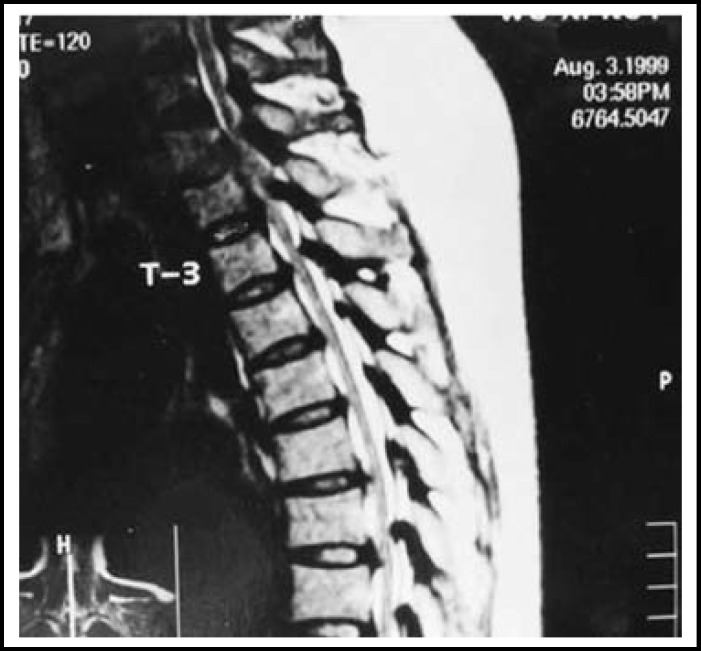
T2 weight MRI of thoracic spine showed multilevel cord compression by the bulging disc between C7 & T9 vertebrae

 Ligamentum flavum were found normal in 3 (11.1%) patients only. It was hypertrophied in 23(85.2%) patients and hypertrophied as well as ossified/calcified in 1(3.7%) patient. In 10(37%) cases only dorsal (thoracic) level were involved, in 3(11.1%) cases only lumbar level were involved and both were involved in 11(40.7%) cases. In 6(%) cases a single level was involved, in 8(%) cases two levels were involved and in 10(%) cases more than 2 levels were involved. The most frequently involved level was D11-D12 seen in 14(%) cases. In 2(%) cases there was a continuous involvement of adjacent vertebrae causing block vertebrae. There was mild thickening of anterior and posterior longitudinal ligaments in 14 (51.8%) and moderate thickening in 08(29.6%) cases (Fig.1). These enlargements were indenting the adjacent epidural sac. (Fig.2)

 Encroachment and narrowing of neural foramina was found in 22 cases. While anterior disc herniation was seen in 74%, lateral disc herniation leading to indentation of thecal sac and narrowing of neural foramina was found in 62.9% cases. These changes were most frequently seen at L4-L5 level in 18(66.7%) cases. In 5 (18.5%) cases these changes were seen L5-S1 in as well as L4-L5.

 Generalized iliac wings’ sclerosis was seen in 19(70.4%) out of 27 patients. In 14 cases it was mild and in 95 cases moderate to severe. Facet joint sclerosis was seen in 5(18.5%) cases (Fig.2). The para- vertebral soft tissue was normal in all cases.

## Discussion

 To the best of authors’ knowledge, this study is the largest series to report the spectrum of MRI features in spinal flourosis some of which have not been reported previously. All of these subjects complained of a vague back ache without localizing neurological signs on physical examination. They had a sclerosed spine on plain X-rays and all showed abnormal findings on dorso- lumbar MRI examination which were rather unusual in appearance. In fact, the originally reporting radiologist considered the changes as that of a hemoglobinopathy till placed in possession of laboratory profile.

 The findings described in literature with reference to flourosis include sclerotic/dense bones, ossified posterior longitudinal ligament and ligamentum flavum, stress fractures, spinal canal and neural foramina narrowing caused by osteophytes in addition to possible presence of menigocoele.^10^^,^^12^

 Osteosclerosis that is increased density of the bones has previously been described on MRI as low signal intensity on both T1 and T2 weighted images.^2^^,^^14^ The same findings were seen in all cases of this series. The presence of MRI signal changes, neural foramina narrowing and presence of premature degeneration of vertebrae in the absence of localizing signs and with nonspecific symptomatology in this series is supported by Assefa et al. who found that radiological findings may be present in many asymptomatic flourotic subjects.^5^

 Although sclerotic bones can be visualized on plain x-rays and CT scan, MRI has a distinct superiority over other modalities in demonstration of intraspinal ligaments particularly when they are not calcified.^10^ This was proven in this series where a vast majority i.e. about 89% subjects exhibited ligamentum flavum hypertrophy and only one had it ossified. On the other hand, ossification/ calcification of the posterior longitudinal ligament and ligamentum flavum which has been reported in literature as the prominent finding,^9^^,^^10^^,^^12^^,^^15^^,^^16^ was not seen in this study. Rather both the anterior and posterior longitudinal ligaments were thickened. This discrepancy can be explained by the difference in the modalities used for imaging the spine; most of the other studies used either plain x-rays or CT scan with less soft tissue evaluation capability than MRI. Also the other reports described an evaluation of the symptomatic subjects while this was almost a screening study of an endemic population where there were minimal symptoms and no signs. MRI is a costly investigation and not feasible for epidemiological studies on endemic population which may be the main reason for paucity of MRI literature on flourosis. The cost of MRI scans in this study was borne by a philanthropist.

 Another probably incidental finding is the presence of multiple hemangiomas in about 15% of the study subjects. Although hemangioma is an uncommon tumor it may be seen as incidental finding in 1.5% cases on MRI scans done for other reasons.^17^ However the frequency was 10 times higher in this study. Perhaps the altered bone structure paved way for development of abnormal vessels’ cluster. This is a new feature not previously reported; the previously reported association was that with pseudomeningocele.^10^ Still this may represent a purely incidental finding as another regional study has mentioned that hemangiomas can be seen as incidentalomas in as much as 26.9% population.^18^

 The main strength of this study is reporting the frequency and extent of pre-symptomatic soft tissues changes that occur in the flourotic spine on an appropriate and sensitive imaging modality. A novel feature of a high frequency of co-existent hemangiomas has been reported for the first time. The main limitation is the cross sectional observational nature of study that prevented deriving hypotheses and follow up changes correlated with symptom onset over an extended period of time.

## Conclusions

 Vertebral sclerosis, a combination of premature degeneration with anterior disc herniation and an unusually high frequency of vertebral hemangioma formed the spectrum of MRI findings in subjects with spinal flourosis having back ache but no neurological findings.

## Notes

***Institute from where the work originates*** *: *

Dow University of Health Sciences Karachi, Baba-e- Urdu Road, Karachi, Pakistan.
